# Clinical, Virological, and Pathological Outcomes Associated with Viral Dose in AG129 Mice Infected with Chikungunya Virus: An In Vivo Model to Study Viral Pathogenesis and Antiviral Preclinical Evaluation

**DOI:** 10.3390/pathogens15050454

**Published:** 2026-04-22

**Authors:** Marília Mazzi Moraes, Natália de Godoy, Eduardo Maffud Cilli, Paulo Ricardo da Silva Sanches

**Affiliations:** 1 Institute of Chemistry, São Paulo State University (UNESP), Araraquara 14800-060, SP, Brazil; marilia.mazzi@unesp.br (M.M.M.); eduardo.cilli@unesp.br (E.M.C.); 2 School of Pharmaceutical Sciences, São Paulo State University (UNESP), Araraquara 14800-903, SP, Brazil; n.godoy@unesp.br

**Keywords:** Chikungunya virus, animal model, viral kinetics, AG129 mice

## Abstract

Chikungunya virus (CHIKV) infection presents a wide spectrum of clinical outcomes, ranging from mild self-limiting disease to severe and fatal manifestations, which are influenced by both host and viral factors. Animal models are essential for elucidating CHIKV pathogenesis and for preclinical evaluation of antiviral strategies; however, a well-characterized model evaluating the effect of different viral doses in AG129 mice remains limited. In this study, we investigated the clinical, virological, and pathological outcomes of CHIKV infection in male AG129 mice inoculated intraperitoneally with different viral doses (10, 100, and 1000 PFU/mL) of a Brazilian strain belonging to the East/Central/South African (ECSA) lineage. Lower-dose inoculation (10 PFU/mL) resulted in a milder disease course, characterized by transient viremia, limited tissue viral dissemination, minimal histopathological alterations, partial survival, and viral clearance. In contrast, higher doses (≥100 PFU/mL) led to rapid systemic viral dissemination, severe histopathological damage in the spleen, liver, and kidneys, and uniform lethality. Viral RNA was detected in serum and multiple organs in a time-dependent manner, with limited differences among inoculum doses in most tissues. Notably, dose-related differences were observed in specific compartments and time points, particularly in hind-limb muscles at early time points and in serum at later stages.

## 1. Introduction

Chikungunya virus (CHIKV) is an *Alphavirus* belonging to the *Togaviridae* family [1]. Its positive-sense single-stranded RNA genome is approximately 11.8 kilobases in length and encodes two polyproteins that are cleaved into nine proteins: four non-structural (nsP1–nsP4) and five structural (C, E1, E2, E3, and 6K) [2]. Based on genomic differences, CHIKV is classified into three genotypes: East/Central/South African (ECSA), West African, and Asian [3].

CHIKV was first isolated in Tanzania in 1952 [4]. The arbovirus is transmitted through the bites of female *Aedes* spp. mosquitoes [2]. The most common symptoms of Alphavirus chikungunya fever include fever, arthralgia, headache, and rash. In some patients, chronic arthralgia can persist for months [5]. Although two vaccines are currently available against CHIKV, there is still no specific antiviral treatment for Alphavirus chikungunya fever [6,7].

Animal models are essential for understanding the pathogenesis and immune responses associated with CHIKV infection [8]. AG129 mice, which lack alpha/beta and gamma interferon receptors (IFN-α/β and INF-γ), are widely used as a murine model for studying arboviruses such as Dengue virus (DENV) and Zika virus (ZIKV) [9,10,11]. A descriptive or well-established animal model for CHIKV infection in AG129 mice has not yet been developed. In this study, we infected AG129 mice with different doses of CHIKV to evaluate the clinical manifestations, viral replication kinetics, and histopathological changes associated with infection. The objective was to establish a descriptive disease profile in this immunocompromised mouse model and to identify a suitable viral dose for use in future antiviral efficacy assays. This approach aimed to provide a reliable and reproducible in vivo model for studying CHIKV pathogenesis and for the preclinical evaluation of therapeutic candidates.

## 2. Materials and Methods

### 2.1. Virus and Titration

The CHIKV used in this study was kindly provided by the Laboratory of Virology Research from the São José do Rio Preto School of Medicine (FAMERP). The virus strain belongs to the ECSA lineage and was originally isolated in Brazil in 2014 (strain BHI3762, accession number H804917) [12]. The virus was cultured in Vero E6 cells using Dulbecco’s Modified Eagle Medium (DMEM) High Glucose (Corning, New York, NY, USA), supplemented with 1% Antibiotic–Antimycotic Solution (Sigma-Aldrich, Saint Louis, MO, USA), 1% of Non-essential Amino Acids and 2% Fetal Bovine Serum (FBS) (Corning, New York, NY, USA).

The viral titter was determined by plaque assay. Vero E6 cells (7 × 10^4^/well) were seeded in 24-well plates (Kasvi, São José dos Pinhais, PR, Brazil). After 24 h, the cells were infected with tenfold serial dilutions of the CHIKV ECSA lineage and incubated in a humidified 5% CO_2_ incubator for 1 h at 37 °C. The inoculum was then removed and replaced with fresh Dulbecco’s Modified Eagle Medium (DMEM) High Glucose (Corning, New York, NY, USA), supplemented with 1% Antibiotic–Antimycotic Solution (Sigma-Aldrich, Saint Louis, MO, USA), 1% of Non-essential Amino Acids (Sigma-Aldrich, Saint Louis, MO, USA), 2% Fetal Bovine Serum (FBS) (Corning, New York, NY, USA), and 1% Carboxymethylcellulose (Merck, Darmstadt, Germany). Infected cells were incubated for 72 h at 37 °C in a humidified incubator with 5% CO_2_. After incubation, the cells were fixed with 10% Formaldehyde (Êxodo Científica, Sumaré, SP, Brazil) and stained with 1% Crystal Violet Solution (Merck, Darmstadt, Germany). Viral plates were counted, and the viral titter was expressed as plaque-forming units per milliliter (PFU/mL).

### 2.2. Ethics Statement and Animals

This study was approved by the Animal Ethics Committee of School of Pharmaceutical Sciences of São Paulo State University (protocol number: 6212832739). Male AG129 mice were sourced from the in-house breeding colony maintained by the Laboratory of Molecular Virology. Animals were housed under controlled environmental conditions (temperature, humidity, and 12 h light/dark cycle) with ad libitum access to food and water. To promote animal welfare, environmental enrichment was provided throughout the experimental period, including nesting material and shelters, allowing the expression of natural behaviors and reducing stress. All procedures were conducted in accordance with national and institutional guidelines for the care and use of laboratory animals. Humane endpoints were predefined, and animals presenting severe clinical signs or excessive weight loss were promptly euthanized to minimize suffering. Mice aged 8–10 weeks were intraperitoneally (IP) inoculated with the CHIKV ECSA lineage. Animals were monitored daily for clinical signs and body weight.

### 2.3. Mice Experiments

To assess differences in CHIKV infection, AG129 mice were IP inoculated with 10, 100, and 1000 PFU/mL of virus. The three viral doses were diluted in 100 µL of Phosphate-Buffered Saline (PBS; pH 7.4, 0.9% NaCl) and administered to each mouse in the three groups. A total of 19 animals per group was used. The same cohort of animals was used for longitudinal evaluation. Serum, spleen, liver, kidney, and hind-limb muscles were collected from a minimum of two animals per group daily for up to seven days post inoculation. When no deaths occurred on a given day, at least two animals were euthanized. Samples were used to determine viral loads at each time point. Because animals were euthanized at predefined time points, the number of animals available for survival and body weight analyses decreased over time. Survival was monitored daily, and body weight was recorded throughout the experiment in the remaining animals until death or euthanasia.

### 2.4. Histopathological Analysis

Spleen, liver, kidney, and hind-limb muscle samples from five mice per group (10, 100, and 1000 PFU/mL) were collected at the time of death for histopathological analysis. Samples from the 1000 PFU/mL group were collected on day 3 post inoculation, whereas samples from the 100 PFU/mL group were collected on days 3 and 4 post inoculation. In the 10 PFU/mL group, samples were collected on days 4, 5, 6, and 7 post-inoculation. As animals in this group remained alive on day 7 post inoculation, they were euthanized at this time point. Tissues were fixed in 10% formaldehyde (Synth, São Paulo, Brazil) for 24 h, followed by dehydration in 70% ethanol (Merck, Darmstadt, Germany) for an additional 24 h. Samples were then embedded in paraffin, sectioned, and stained with hematoxylin and eosin (H&E) for histopathological evaluation. All analyses were performed by a blinded pathologist.

### 2.5. RNA Quantification

Prior to RNA extraction, tissues were resuspended in 500 μL of PBS and homogenized using the FastPrep-24™ 5G (MP Biomedicals, Santa Ana, CA, USA). Viral RNA was extracted from 140 μL of tissue homogenates and serum using TRIzol reagent (Invitrogen, Carlsbad, CA, USA), according to the manufacturer’s instructions. RNA levels were quantified by RT-qPCR using the GoTaq^®^ Probe 1-Step RT-qPCR System (Promega, Madison, WI, USA), following the manufacturer’s protocol. Primers and probes were based on Lanciotti et al., 2007 [13]. Standard curves were generated by the RT-qPCR instrument software using serial dilutions and used for viral RNA quantification. Results were expressed as log10-transformed values. All samples were processed under the same experimental conditions, using a fixed volume of serum or tissue homogenate for RNA extraction and RT-qPCR. Standard curves are shown in Supplementary Figure S1.

### 2.6. Statistical Analysis

Statistical analyses were performed using GraphPad Prism (v8.0.2, 2019; San Diego, CA, USA). Viral RNA levels were log10-transformed prior to analysis. Comparisons among multiple groups were performed using one-way ANOVA followed by Tukey’s multiple comparisons test. For comparisons between two groups, unpaired t-tests with Welch’s correction were applied. Statistical significance was set at *p* < 0.05.

## 3. Results

### 3.1. AG129 Mice Response to CHIKV Infection

**Figure 1:**
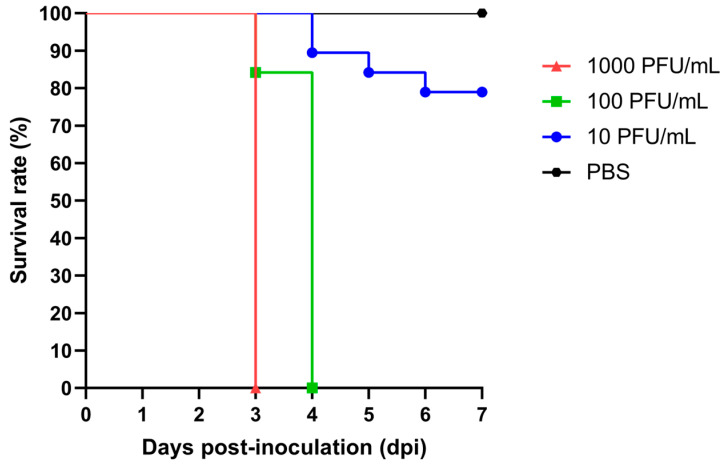
Survival rate of AG129 mice control group and AG129 mice IP inoculated with 10, 100, and 1000 PFU/mL of CHIKV. The control group comprised 6 mice, and experimental groups initially comprised 19 mice each. Due to scheduled euthanasia for sample collection, the number of animals decreased over time, and survival was calculated based on the remaining animals at each time point.

**Figure 2:**
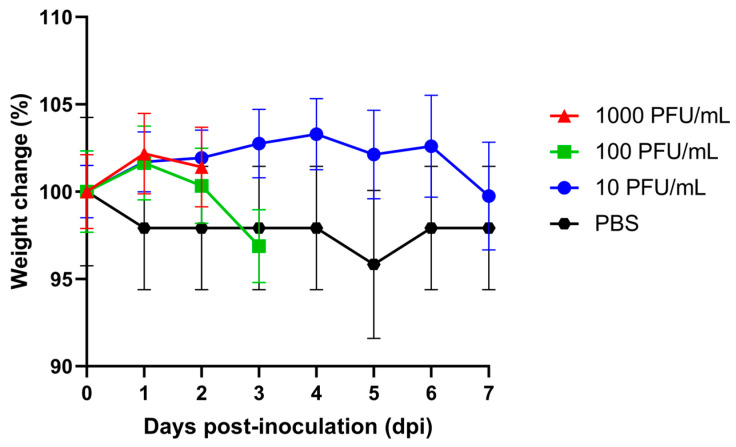
Average body weight of AG129 mice control group and AG129 mice IP inoculated with 10, 100, and 1000 PFU/mL of CHIKV. The control group comprised 6 mice, and experimental groups initially comprised 19 mice each. Due to scheduled euthanasia for sample collection, the number of animals decreased over time.

When inoculated intraperitoneally with 10 PFU/mL, AG129 mice developed signs of illness such as lethargy, squinted eyes, and retracted ears from day 3 to day 6 post inoculation. During the observation period, 78.95% of the animals survived, and their average body weight remained relatively stable (Figure 1 and Figure 2). In contrast, when inoculated intraperitoneally with 100 and 1000 PFU/mL, 0% of the AG129 mice survived after 4 and 3 days post inoculation, respectively. Mice inoculated with 100 PFU/mL showed signs of illness, including lethargy and weight loss, starting on day 3 post inoculation. Interestingly, none of the animals inoculated with 1000 PFU/mL of CHIKV displayed visible signs of illness and instead showed a slight increase in average body weight during the observation period. The control group, which was IP inoculated with PBS, presented a slight loss of average body weight but did not develop any signs of illness during the observation period (Figure 1 and Figure 2).

### 3.2. CHIKV RNA Kinetics in AG129 Mice Serum and Tissues

Viral RNA was detected in serum and different organs, indicating systemic dissemination of CHIKV. However, quantitative analysis showed that viral RNA levels were generally similar among the inoculum groups in most tissues, especially at early time points. No significant differences were observed in kidney, liver, and spleen at day 3 and day 4 post inoculation, although small variations among groups were observed at day 3.

In contrast, hind-limb muscles presented significant differences at day 3, suggesting an early tissue-specific response related to the viral dose. In serum, differences were observed at day 4 post inoculation; however, comparisons were limited due to the absence of data from all inoculum groups at this time point.

Overall, these results indicate that CHIKV spreads systemically across tissues, but the effects of the inoculum dose are not uniform and appear to be restricted to specific tissues and time points.

### 3.3. Organ Histopathology of AG129 Mice After Infection with Different Doses of CHIKV

In animals inoculated with 10 PFU/mL, the spleen showed mild reactive lymphoid hyperplasia with slight vascular alterations, the liver exhibited mild reactive hepatopathy with some Kupffer cell activation, minimal inflammation and sinusoidal congestion, and the kidneys were largely preserved with only focal mild changes. Animals inoculated with 100 PFU/mL presented more evident alterations, including follicular hyperplasia and white pulp expansion in the spleen, with congestion and hemorrhage in the red pulp and macrophage activation. In the liver, Kupffer cell activation and moderate histopathological changes were observed, while the kidneys showed mild to moderate tubular injury with congestion and slight inflammation.

The most extensive lesions were observed in animals inoculated with 1000 PFU/mL, including marked splenic alterations with white pulp expansion, congestion and hemorrhage, as well as macrophage activation. In the liver, diffuse lesions were observed, including congestion, sinusoidal hemorrhage, and necrosis. The kidneys presented moderate acute tubular injury associated with interstitial hemorrhage, congestion, and mild inflammation.

**Figure 3:**
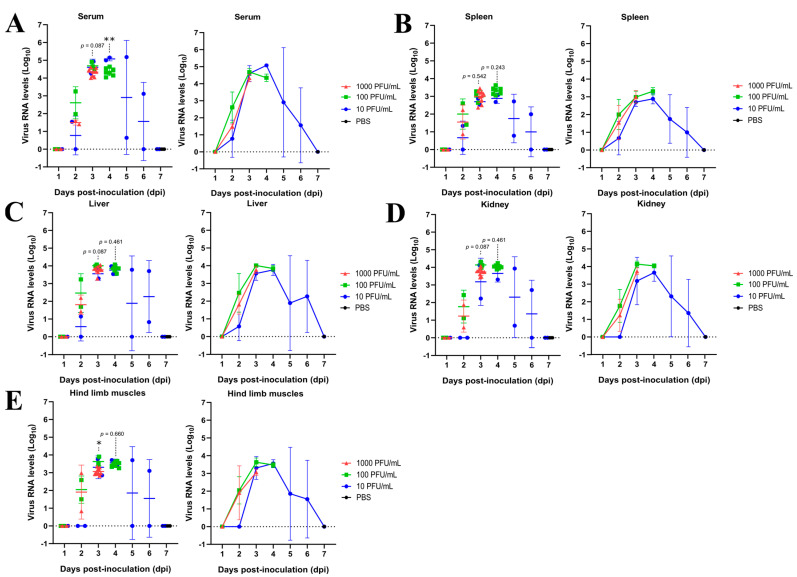
Viral RNA levels in serum and tissues of CHIKV-infected AG129 mice. Viral RNA levels (log_10_) were measured by RT-qPCR in serum (**A**), spleen (**B**), liver (**C**), kidney (**D**), and hind-limb muscles (**E**) of mice infected with 10, 100, or 1000 PFU/mL. Data are shown as mean ± SD. A significant difference was observed in hind-limb muscles at day 3 (* *p* = 0.0167, one-way ANOVA with Tukey’s test). In serum at day 4, a significant difference was observed between available groups (** *p* = 0.0035, unpaired *t*-test with Welch’s correction), as not all inoculum groups were represented at this time point. No significant differences were detected in the other tissues. PBS was used as control. Dotted lines indicate the limit of detection.

**Figure 4:**
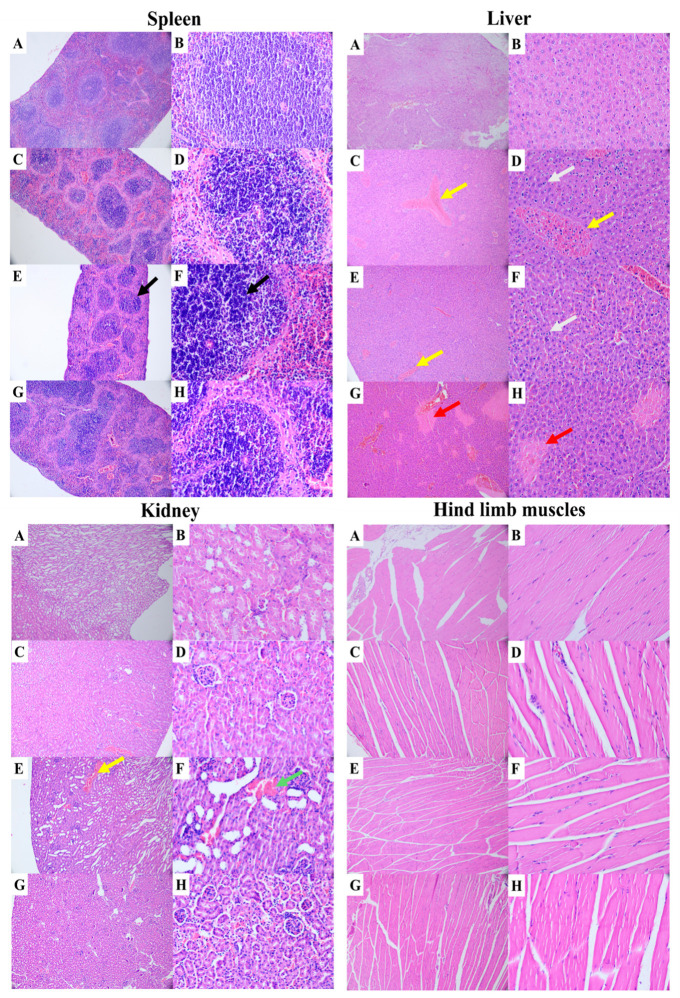
Histopathological analysis of the spleen, liver, kidney, and hind-limb skeletal muscle of AG129 mice. Control group is shown in panels (**A**,**B**), and AG129 mice inoculated IP with CHIKV at doses of 10 PFU/mL (**C**,**D**), 100 PFU/mL (**E**,**F**), and 1000 PFU/mL (**G**,**H**). Black arrows indicate lymphoid hyperplasia; yellow arrows indicate congestion; green arrows indicate hemorrhage; white arrows indicate inflammatory infiltrates; and red arrows indicate necrosis. Scale bars correspond to 10× (left panels) and 40× (right panels) magnification.

No significant histopathological alterations were observed in hind-limb muscles across the inoculated groups, despite the detection of viral RNA in this tissue (Figure 3 and Figure 4, Table 1). RT-qPCR analysis showed that viral RNA levels in hind-limb muscles were generally lower than those observed in visceral organs, particularly at early time points. These findings suggest that, although CHIKV reaches muscle tissue, viral replication in this compartment may be limited or insufficient to induce detectable structural damage.

Importantly, although histopathological alterations appeared more pronounced with increasing inoculum doses in visceral organs, these differences were not statistically supported by viral RNA quantification, indicating that dose-dependent effects were not consistently observed in these tissues.

## 4. Discussion

Murine models are widely used in viral research due to their compact physiology, experimental efficiency, and ease of genetic manipulation [14]. The clinical manifestations of CHIKV infection are influenced by host factors, such as age and immune status, as well as viral parameters, including strain and route of inoculation [15]. The interferon response is critical for protection against CHIKV, particularly during the acute phase of infection [16,17,18]. In this context, we established an experimental model using 8–10 weeks age male AG129 mice, which lack both type I and II interferon receptors (IFN-α/β and IFN-γ), inoculated intraperitoneally with the ECSA lineage, currently prevalent strain in Brazil.

Evaluating different CHIKV doses in AG129 mice was important to reproduce the spectrum of disease outcomes observed in humans. Although CHIKV infection is typically self-limiting, severe manifestations are mainly reported in children, elderly individuals, and patients with underlying comorbidities [19,20,21,22,23]. Viral RNA levels became detectable on day 2 post infection in the serum of the three AG129 mice groups infected with different CHIKV doses and increased up to day 4 post inoculation, consistent with the reported incubation period of the virus in humans [24]. Detection of viral RNA in the spleen, kidney, liver, and hind-limb muscles from day 3 post inoculation onward across all three groups confirms systemic viral dissemination, as previously reported in murine model studies of arboviruses [9,10,11,15] (Figure 3, Table 1). Notably, mice inoculated with 10 PFU/mL maintained stable average body weight and exhibited transient viral RNA levels in serum and tissues, followed by recovery, reflecting a mild disease course. In contrast, higher inoculation doses (≥100 PFU/mL) led to weight changes, severe disease, and mortality (Figure 1 and Figure 2, Table 1).

To further explore the inoculum dose effects observed, we analyzed viral RNA kinetics across tissues and time points. On day 3 post inoculation, comparable viral RNA levels were detected in the serum, spleen, liver, kidney, and hind-limb muscles across the three AG129 mouse groups. However, animals inoculated with 10 PFU/mL displayed lower viral RNA levels in serum and tissues at days 1 and 2 post inoculation compared with those inoculated with 100 or 1000 PFU/mL, suggesting delayed or limited early viral replication at lower doses (Figure 3, Table 1). In contrast, eight-week-old AG129 mice inoculated via the hind footpad with increasing doses of ZIKV developed comparable viremia levels regardless of the inoculum, culminating in uniform lethality by day 8 post inoculation [11]. Overall, viral RNA levels were comparable among groups in most tissues and time points. A statistically significant difference was observed in hind-limb muscle at day 3 post infection (one-way ANOVA with Tukey’s multiple comparisons test, * *p* = 0.0167), indicating a transient dose-dependent effect in this tissue. In serum at day 4, where only two groups remained available, a significant difference was detected using an unpaired t-test with Welch’s correction (** *p* = 0.0035). No significant differences were observed in spleen, liver, or kidney at any evaluated time point.

The spleen is a key site for arboviruses replication and immune modulation; these viruses infect splenic cell populations, such as monocytes and macrophages, and induce the production of pro-inflammatory cytokines and type I interferons (INF-I) [25,26,27]. Histopathological analysis of the spleen from the three groups inoculated with different doses of CHIKV revealed follicular hyperplasia, expansion of the white pulp, and congestion of the red pulp, reflecting lymphoid activation following virus infection. In the livers, histopathological changes appeared more pronounced with increasing CHIKV doses. Alterations in the livers of animals inoculated with 1000 PFU/mL of the virus included sinusoidal congestion, Kupffer cell activation, portal inflammatory infiltrates, and hepatocytes displaying swelling, focal degeneration, and necrosis, a pattern consistent with systemic inflammatory responses and a possible indirect cytokine-mediated injury [28,29]. The kidney of the three groups inoculated with CHIKV exhibited diffuse congestion, tubular swelling and degeneration, focal necrosis, and interstitial lymphocytic infiltration, findings compatible with acute kidney injury induced by both direct viral damage and, predominantly, intense systemic inflammation and endothelial dysfunction—mechanisms previously described in animal models and in severe human cases of CHIKV disease [30,31,32,33,34,35,36] (Figure 3 and Figure 4, Table 1).

The presence of viral RNA in the absence of histopathological changes may reflect transient viral dissemination, low-level infection of specific cell populations (e.g., muscle satellite cells), or persistence of residual viral RNA without productive infection. The absence of histopathological alterations in hind-limb muscle, despite detectable viral RNA, can be explained by several factors intrinsic to this experimental model. First, AG129 mice lack functional type I and type II interferon signaling pathways, which play a central role not only in antiviral defense but also in shaping immune-mediated tissue damage. As a result, the inflammatory responses typically associated with alphavirus-induced muscle pathology may be altered or insufficient to produce detectable structural lesions. Second, the rapid disease progression observed at higher inoculum doses, culminating in early lethality, likely limits the time required for the development of overt tissue damage in peripheral compartments such as skeletal muscle. Finally, the detection of viral RNA does not necessarily indicate productive infection, as it may reflect residual viral genomes, limited replication, or transient viral dissemination without effective amplification within the tissue. Together, these factors support the interpretation that viral presence in muscle is not sufficient to drive tissue injury in this model, highlighting the importance of both viral replication dynamics and host immune responses in determining pathological outcomes.

Despite the clear differences in viral load observed during the early days post infection, the data demonstrate that the maximum peak viral load is similar across all inoculated viral doses (3–4 days post inoculation) (Figure 3). This finding suggests that, once systemic infection is established, CHIKV replication reaches a common biological limit, likely determined by the maximal replicative capacity of target tissues and the availability of permissive cells. This behavior is consistent with a saturable viral replication model, in which factors such as limited availability of susceptible cellular niches, intracellular viral competition, and the activation of residual innate immune responses impose an upper limit on viral replication [37]. Similar patterns have been reported in other arthritogenic alphaviruses related to CHIKV, such as Ross River virus and Sindbis virus, in which peak viremia converges despite differences in inoculum dose. These studies indicate that disease severity is more strongly influenced by early viral replication kinetics rather than absolute peak viral load [25,26,34]. Accordingly, our findings reinforce that early viral expansion plays a critical role in determining clinical outcome, as animals exposed to higher inoculum doses experience a more intense and sustained early viral burden, which may trigger exacerbated inflammatory responses and irreversible tissue damage prior to reaching peak viral levels. However, despite similar peak viral loads across groups, our results also revealed tissue- and time-specific differences, particularly in hind-limb muscles at early time points and in serum at later stages, indicating that viral dynamics are not uniform across compartments.

Taken together, these findings suggest that CHIKV pathogenesis in AG129 mice is not governed by a simple dose–response relationship but rather by complex interactions between viral replication kinetics, tissue-specific susceptibility, and host responses.

## 5. Conclusions

In this study, we established an animal model to study the effects of different Chikungunya virus (CHIKV) inoculum doses in AG129 mice. Infection with 10 PFU/mL resulted in a milder and self-limiting disease, with delayed viral replication, limited tissue dissemination, and partial survival. In contrast, higher doses (≥100 PFU/mL) led to rapid disease progression, systemic viral spread, and uniform lethality.

Our findings indicate that disease severity is influenced by early viral replication dynamics rather than peak viral load alone, highlighting the importance of early viral expansion in determining clinical outcome. Additionally, the dissociation between viral RNA detection and tissue damage in hind-limb muscles suggests that viral presence alone is not sufficient to induce tissue injury.

From a translational perspective, the 10 PFU/mL condition provides a useful experimental window that allows measurable viral replication while maintaining host survival, supporting its application in future antiviral evaluation studies. Differences observed in specific tissues and time points, such as hind-limb muscles at day 3 and serum at day 4, indicate that viral dynamics are not uniform across compartments.

Overall, this model contributes to the understanding of CHIKV pathogenesis and provides a platform for preclinical investigation.

## Figures and Tables

**Table 1:** Summary of viral replication kinetics, tissue dissemination, clinical progression, and histopathological outcomes in AG129 mice infected with CHIKV at different inoculum doses.

CHIKV Dose (PFU/mL)	Viral Replication Kinetics	Peak Viral RNA (dpi)	Tissue Dissemination	Clinical Outcome	Histopathological Severity	Survival
10	Delayed and gradual	4	Limited, transient	Mild disease	Minimal to mild	Partial
100	Rapid and intense	3–4	Systemic	Severe disease	Moderate to severe	0%
1000	Very rapid and early	3	Systemic	Severe disease	Severe	0%

## Data Availability

We declare that the main data supporting our findings are available within the article.

## Supplementary Materials

The following supporting information can be downloaded at: https://www.mdpi.com/article/10.3390/pathogens15050454/s1, Figure S1: Standard curves used for CHIKV RNA quantification by RT-qPCR. Serial dilutions were used to generate standard curves for each viral concentration (10, 100, and 1000 PFU/mL) (A–C). The curves were generated by the RT-qPCR instrument software and used to estimate viral RNA levels in the samples.

## References

[1] Strauss J.H., Strauss E.G. (1994). The alphaviruses: Gene expression, replication, and evolution. Microbiol. Rev..

[2] Pialoux G., Gaüzère B.A., Jauréguiberry S., Strobel M. (2007). Chikungunya, an epidemic arbovirosis. Lancet Infect. Dis..

[3] Weaver S.C. (2014). Arrival of chikungunya virus in the new world: Prospects for spread and impact on public health. PLoS Neglected Trop. Dis..

[4] Ross R.W. (1956). A laboratory technique for studying the insect transmission of animal viruses, employing a bat-wing membrane, demonstrated with two African viruses. Epidemiol. Infect..

[5] Rama K., de Roo A.M., Louwsma T., Hofstra H.S., Gurgel do Amaral G.S., Vondeling G.T., Postma M.J., Freriks R.D. (2024). Clinical outcomes of chikungunya: A systematic literature review and meta-analysis. PLoS Neglected Trop. Dis..

[6] Schneider M., Narciso-Abraham M., Hadl S., McMahon R., Toepfer S., Fuchs U., Hochreiter R., Bitzer A., Kosulin K., Larcher-Senn J. (2023). Safety and immunogenicity of a single-shot live-attenuated chikungunya vaccine: A double-blind, multicentre, randomised, placebo-controlled, phase 3 trial. Lancet.

[7] Richardson J.S., Anderson D.M., Mendy J., Tindale L.C., Muhammad S., Loreth T., Tredo S.R., Warfield K.L., Ramanathan R., Caso J.T. (2025). Chikungunya virus virus-like particle vaccine safety and immunogenicity in adolescents and adults in the USA: A phase 3, randomised, double-blind, placebo-controlled trial. Lancet.

[8] Haese N.N., Broeckel R.M., Hawman D.W., Heise M.T., Morrison T.E., Streblow D.N. (2016). Animal models of chikungunya virus infection and disease. J. Infect. Dis..

[9] Milligan G.N., Sarathy V.V., Infante E., Li L., Campbell G.A., Beatty P.R., Harris E., Barrett A.D., Bourne N. (2015). A dengue virus type 4 model of disseminated lethal infection in AG129 mice. PLoS ONE.

[10] Sarathy V.V., White M., Li L., Gorder S.R., Pyles R.B., Campbell G.A., Milligan G.N., Bourne N., Barrett A.D. (2015). A lethal murine infection model for dengue virus 3 in AG129 mice deficient in type I and II interferon receptors leads to systemic disease. J. Virol..

[11] Aliota M.T., Caine E.A., Walker E.C., Larkin K.E., Camacho E., Osorio J.E. (2016). Characterization of lethal Zika virus infection in AG129 mice. PLoS Neglected Trop. Dis..

[12] Nunes M.R., Faria N.R., de Vasconcelos J.M., Golding N., Kraemer M.U., de Oliveira L.F., Azevedo R.D., da Silva D.E., da Silva E.V., da Silva S.P. (2015). Emergence and potential for spread of Chikungunya virus in Brazil. BMC Med..

[13] Lanciotti R.S., Kosoy O.L., Laven J.J., Panella A.J., Velez J.O., Lambert A.J., Campbell G.L. (2007). Chikungunya virus in US travelers returning from India, 2006. Emerg. Infect. Dis..

[14] Zou M., Zheng Z., Wang W., Liu H. (2025). Advances and perspectives for animal models of chikungunya virus infection. Biosaf. Health.

[15] Graham V.A., Easterbrook L., Rayner E., Findlay-Wilson S., Flett L., Kennedy E., Fotheringham S., Kempster S., Almond N., Dowall S. (2024). Comparison of Chikungunya Virus-Induced Disease Progression and Pathogenesis in Type-I Interferon Receptor-Deficient Mice (A129) and Two Wild-Type (129Sv/Ev and C57BL/6) Mouse Strains. Viruses.

[16] Venugopalan A., Ghorpade R.P., Chopra A. (2014). Cytokines in acute chikungunya. PLoS ONE.

[17] Haist K.C., Burrack K.S., Davenport B.J., Morrison T.E. (2017). Inflammatory monocytes mediate control of acute alphavirus infection in mice. PLoS Pathog..

[18] de Souza W.M., Lecuit M., Weaver S.C. (2025). Chikungunya virus and other emerging arthritogenic alphaviruses. Nat. Rev. Microbiol..

[19] de Lima S.T., de Souza W.M., Cavalcante J.W., da Silva Candido D., Fumagalli M.J., Carrera J.P., Simões Mello L.M., De Carvalho Araújo F.M., Cavalcante Ramalho I.L., de Almeida Barreto F.K. (2021). Fatal outcome of chikungunya virus infection in Brazil. Clin. Infect. Dis..

[20] Frutuoso L.C., Freitas A.R., Cavalcanti L.P., Duarte E.C. (2020). Estimated mortality rate and leading causes of death among individuals with chikungunya in 2016 and 2017 in Brazil. Rev. Soc. Bras. Med. Trop..

[21] Bartholomeeusen K., Daniel M., LaBeaud D.A., Gasque P., Peeling R.W., Stephenson K.E., Ng L.F., Ariën K.K. (2023). Chikungunya fever. Nat. Rev. Dis. Primers.

[22] Cerqueira-Silva T., Pescarini J.M., Cardim L.L., Leyrat C., Whitaker H., de Brito C.A., Brickley E.B., Barral-Netto M., Barreto M.L., Teixeira M.G. (2024). Risk of death following chikungunya virus disease in the 100 Million Brazilian Cohort, 2015–2018: A matched cohort study and self-controlled case series. Lancet Infect. Dis..

[23] Pedí V.D., Porto D.L., de Jesus Martins W., de França G.V. (2025). Epidemiology of Chikungunya Hospitalizations, Brazil, 2014–2024. Emerg. Infect. Dis..

[24] Rudolph K.E., Lessler J., Moloney R.M., Kmush B., Cummings D.A. (2014). Incubation periods of mosquito-borne viral infections: A systematic review. Am. J. Trop. Med. Hyg..

[25] Assunção-Miranda I., Cruz-Oliveira C., Da Poian A.T. (2013). Molecular mechanisms involved in the pathogenesis of alphavirus-induced arthritis. BioMed Res. Int..

[26] Guerrero-Arguero I., Tellez-Freitas C.M., Weber K.S., Berges B.K., Robison R.A., Pickett B.E. (2021). Alphaviruses: Host pathogenesis, immune response, and vaccine & treatment updates. J. Gen. Virol..

[27] Rathore A.P., Mantri C.K., Tan M.W., Shirazi R., Nishida A., Aman S.A., Morrison J., St. John A.L. (2021). Immunological and pathological landscape of dengue serotypes 1-4 infections in immune-competent mice. Front. Immunol..

[28] Ribeiro Y.P., Falcão L.F., Smith V.C., de Sousa J.R., Pagliari C., Franco E.C., Cruz A.C., Chiang J.O., Martins L.C., Nunes J.A. (2023). Comparative analysis of human hepatic lesions in dengue, yellow fever, and chikungunya: Revisiting histopathological changes in the light of modern knowledge of cell pathology. Pathogens.

[29] Wang B., Wang Z. (2025). A Review of Arboviral-Induced Liver Damage: Acute Manifestations and Speculative Links to Chronic Inflammation. Rev. Med. Virol..

[30] de Souza W.M., Fumagalli M.J., de Lima S.T., Parise P.L., Carvalho D.C., Hernandez C., de Jesus R., Delafiori J., Candido D.S., Carregari V.C. (2024). Pathophysiology of chikungunya virus infection associated with fatal outcomes. Cell Host Microbe.

[31] Renault P., Solet J.L., Sissoko D., Balleydier E., Larrieu S., Filleul L., Lassalle C., Thiria J., Rachou E., de Valk H. (2007). A major epidemic of chikungunya virus infection on Reunion Island, France, 2005–2006. Am. J. Trop. Med. Hyg..

[32] Economopoulou A., Dominguez M., Helynck B., Sissoko D., Wichmann O., Quenel P., Germonneau P., Quatresous I. (2009). Atypical Chikungunya virus infections: Clinical manifestations, mortality and risk factors for severe disease during the 2005–2006 outbreak on Reunion. Epidemiol. Infect.

[33] Ozden S., Huerre M., Riviere J.-P., Coffey L.L., Afonso P.V., Mouly V., de Monredon J., Roger J.-C., El Amrani M., Yvin J.-L. (2007). Human Muscle Satellite Cells as Targets of Chikungunya Virus Infection. PLoS ONE.

[34] Gardner J., Anraku I., Le T.T., Larcher T., Major L., Roques P., Schroder W.A., Higgs S., Suhrbier A. (2010). Chikungunya virus arthritis in adult wild-type mice. J. Virol..

[35] Teo TeckHui T.T., Lum FokMoon L.F., Claser C., Lulla V., Lulla A., Merits A., Rénia L., Ng L.F. (2013). A pathogenic role for CD4+ T cells during Chikungunya virus infection in mice. J. Immunol..

[36] Filippone C., Legros V., Jeannin P., Choumet V., Butler-Browne G., Zoladek J., Mouly V., Gessain A., Ceccaldi P.E. (2020). Arboviruses and muscle disorders: From disease to cell biology. Viruses.

[37] Pawelek K.A., Huynh G.T., Quinlivan M., Cullinane A., Rong L., Perelson A.S. (2012). Modeling within-host dynamics of influenza virus infection including immune responses. PLoS Comput. Biol..

