# Supplementary material for: Clinical, Virological, and Pathological Outcomes Associated with Viral Dose in AG129 Mice Infected with Chikungunya Virus: An In Vivo Model to Study Viral Pathogenesis and Antiviral Preclinical Evaluation
Source: Pathogens. 2026 Apr 22;15(5):454. doi: 10.3390/pathogens15050454 (PMC13209420; doi:10.3390/pathogens15050454)
Supplement: Supplementary file 1 [file pathogens-15-00454-s001.zip › pathogens-4213688-supplementary.pdf]

pathogens

MDPI

Supplementary Material

A

B

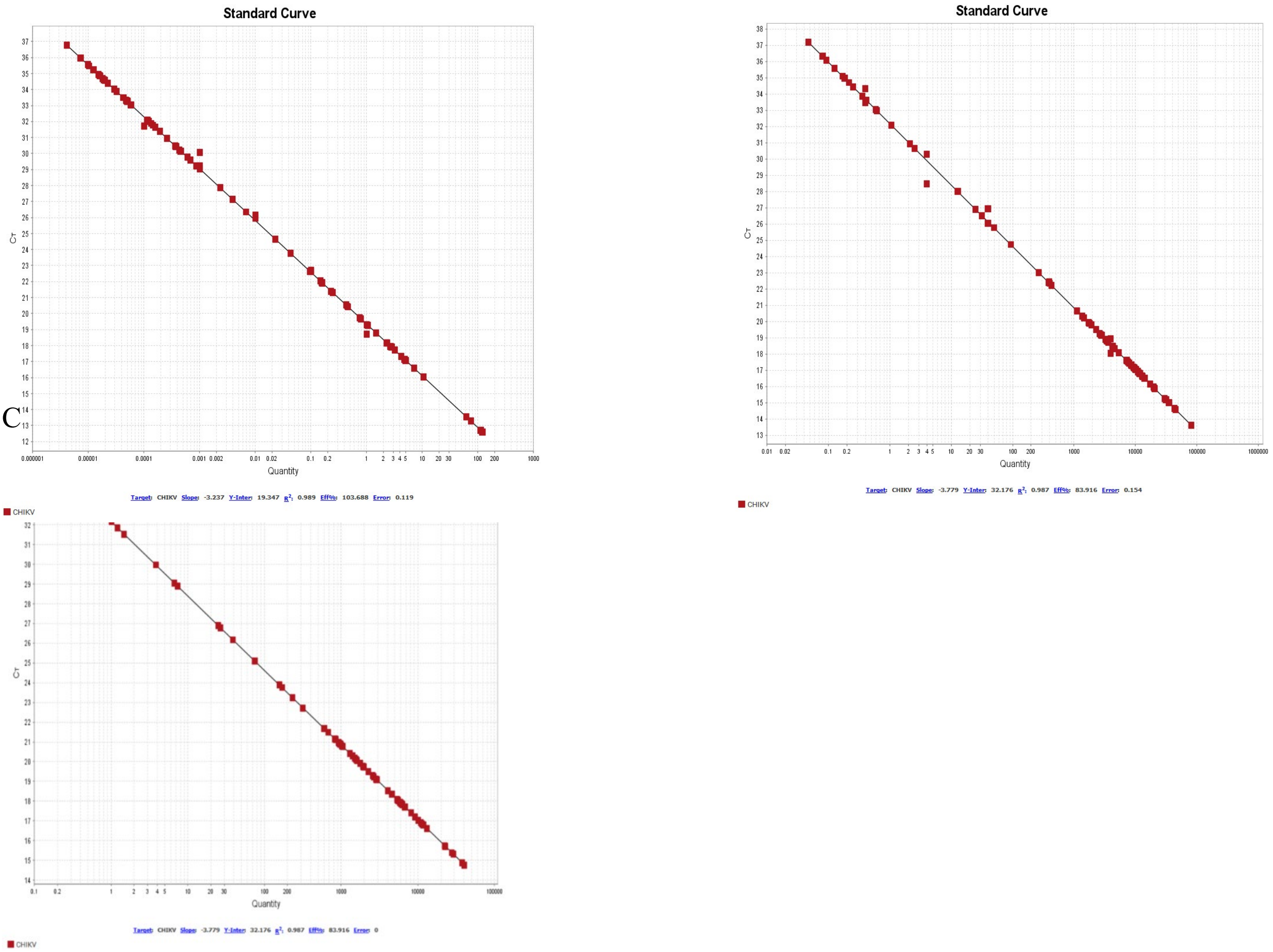

C

Figure S1. Standard curves used for CHIKV RNA quantification by RT-qPCR. Serial dilutions were used to generate standard curves for each viral concentration (10, 100, and 1000 PFU/mL) (A–C). The curves were generated by the RT-qPCR instrument software and used to estimate viral RNA levels in the samples.
